# Food-Like Growth Conditions Support Production of Active Vitamin B12 by *Propionibacterium freudenreichii* 2067 without DMBI, the Lower Ligand Base, or Cobalt Supplementation

**DOI:** 10.3389/fmicb.2017.00368

**Published:** 2017-03-08

**Authors:** Paulina Deptula, Bhawani Chamlagain, Minnamari Edelmann, Panchanit Sangsuwan, Tuula A. Nyman, Kirsi Savijoki, Vieno Piironen, Pekka Varmanen

**Affiliations:** ^1^Department of Food and Environmental Sciences, University of HelsinkiHelsinki, Finland; ^2^Proteomics Unit, Institute of Biotechnology, University of HelsinkiHelsinki, Finland

**Keywords:** *Propionibacterium freudenreichii*, vitamin B12, cobalamin, pseudovitamin B12, food, 2D-PAGE, stress

## Abstract

*Propionibacterium freudenreichii* is a traditional dairy bacterium and a producer of short chain fatty acids (propionic and acetic acids) as well as vitamin B12. In food applications, it is a promising organism for *in situ* fortification with B12 vitamin since it is generally recognized as safe (GRAS) and it is able to synthesize biologically active form of the vitamin. In the present study, vitamin B12 and pseudovitamin biosynthesis by *P. freudenreichii* was monitored by UHPLC as a function of growth in food-like conditions using a medium mimicking cheese environment, without cobalt or 5,6-dimethylbenzimidazole (DMBI) supplementation. Parallel growth experiments were performed in industrial-type medium known to support the biosynthesis of vitamin B12. The production of other key metabolites in the two media were determined by HPLC, while the global protein production was compared by gel-based proteomics to assess the effect of growth conditions on the physiological status of the strain and on the synthesis of different forms of vitamin. The results revealed distinct protein and metabolite production, which reflected the growth conditions and the potential of *P. freudenreichii* for synthesizing nutritionally relevant amounts of active vitamin B12 regardless of the metabolic state of the cells.

## Introduction

*Propionibacterium freudenreichii* is a dairy-associated bacterium, traditionally used in the production of Swiss type cheeses and also for industrial-scale production of vitamin B12 (Martens et al., 2002). The discovery of its probiotic and bifidogenic properties (Kaneko et al., 1994; Mantere-Alhonen, 1995) lead to increased number of studies focusing on the species. As a result, the first genome sequence of the *P. freudenreichii* CIRM-BIA1^T^ was published (Falentin et al., 2010) and then followed by further studies providing deeper insight into the adaptation mechanisms and long-term survival of this species (Thierry et al., 2011; Dalmasso et al., 2012a; Aburjaile et al., 2016).

In the last decade, the natural fortification of foods with vitamins by fermentation with food grade bacteria has been explored (Burgess et al., 2009; LeBlanc et al., 2011; Capozzi et al., 2012; Patel et al., 2013). This method, used to increase the nutritional value of food products without increasing production costs, allows consumers to enhance their vitamin intakes from their normal diet (LeBlanc et al., 2011) and eliminates the need for food supplementation with chemically synthesized vitamin preparations (Capozzi et al., 2012). Cobalamin, also known as vitamin B12, is industrially produced by microbial fermentation, as chemical synthesis is very costly (Martens et al., 2002; Burgess et al., 2009). In addition to *P. freudenreichii* (Van Wyk et al., 2011; Edelmann et al., 2016), also lactic acid bacteria like *Lactobacillus reuteri* (Santos et al., 2008; Molina et al., 2012; Gu et al., 2015) have been utilized in attempts to increase the vitamin B12 levels in food products through fermentation. While several *Lactobacillus* species, including *L. reuteri* (Taranto et al., 2003), *L. plantarum* (Bhushan et al., 2017), and *L. rossiae* (De Angelis et al., 2014), have been shown to produce vitamin B12-like compounds, their ability to produce active vitamin B12 in nutritionally relevant amounts remains to be shown. The reports of *Lactobacillus* studies utilizing methods capable of distinguishing between the different B12 forms are still rare and have revealed that only pseudovitamin B12 is produced (Santos et al., 2007; Crofts et al., 2013). Pseudovitamin B12 differs from the active form by the presence of adenine in the place of 5,6-dimethylbenzimidazole (DMBI) as the lower ligand and it is inactive in humans (Stupperich and Nexø, 1991; Watanabe et al., 2013). In *P. freudenreichii* DMBI is synthesized and activated by recently characterized BluB/CobT2 fusion enzyme (Deptula et al., 2015), whereas the ability of Lactobacilli to synthesize DMBI has yet to be demonstrated.

Cobalt and DMBI are frequently added for industrial production of vitamin B12, since they are necessary to form the corrin ring and the lower ligand of the molecule, respectively, and are considered the limiting factors in vitamin B12 synthesis (Marwaha et al., 1983; Martens et al., 2002; Hugenschmidt et al., 2011). Neither of these substrates, however, is allowed in food applications and strategies for food fortification without these additions are needed. Among the microorganisms typically used for the production of vitamin B12, *P. freudenreichii* is the only food grade bacterium with known capability to synthesize DMBI and it is becoming a viable candidate for *in situ* vitamin B12 production during food fermentation.

In Swiss-type cheese, *P. freudenreichii* essentially grows in the absence of carbohydrates, since the milk lactose is efficiently fermented to lactic acid by lactic acid bacteria. Swiss-type cheese is rich in soluble nitrogen that, with lactic acid as the carbon source, supports the growth of Propionibacteria. Thus, a laboratory growth medium based on sodium lactate and a nitrogen source, such as yeast extract, has been used to mimic nutritional conditions for *P. freudenreichii* in cheese (Dalmasso et al., 2012a,b). Although, Propionibacteria grow poorly in milk and milk whey inhibits their growth (Piveteau et al., 2000), a whey-based medium supplemented with yeast extract has often been used for industrial vitamin B12 production (Bullerman and Berry, 1966; Marwaha et al., 1983; Hugenschmidt et al., 2011).

Here we studied the production of active vitamin B12 and pseudovitamin B12 by a *P. freudenreichii* strain in a food-like environment in a medium mimicking cheese conditions, without cobalt and/or DMBI supplementation. For comparative analyses, the strain was also propagated in an industrial-type whey medium known to support the production of active B12 vitamin by the studied strain (Chamlagain et al., 2016). In parallel, the production of other key metabolites and the global protein production by *P. freudenreichii* in these two conditions was also monitored to elucidate the effect of different growth conditions, and the different physiological statuses of the strain, on its ability to produce active vitamin B12, with future food applications in mind.

## Materials and methods

The strain of *P. freudenreichii* 2067, was obtained from Valio Ltd. and assigned to the subspecies *shermanii* based on 100% identity of the 16S RNA partial sequence (see Supplemental File 1) with the published genome of *P. freudenreichii* CIRM-BIA1^T^ (Falentin et al., 2010).

### Growth conditions and media

The growth media used were a cheese-like propionic medium (PPA), and the industrial-type whey-based liquid medium (WBM). The PPA composition was: 5.0 g. tryptone (Sigma-Aldrich), 10.0 g of yeast extract (Becton, Dickinson), and 14.0 ml 60% w/v DL-sodium lactate (Sigma-Aldrich) per liter (pH of the medium was adjusted to 6.7 prior to autoclaving; Suomalainen et al., 2008). The industrial-type medium, WBM, was composed of 60.0 g of filtered whey powder (Valio Ltd., Finland), 10.0 g of yeast extract (MERCK, KGaA), 13 g of sodium D/L-lactate syrup 60% w/w (Sigma-Aldrich), 0.1 g of Tween 80 (MERCK, KGaA), 0.2 g of magnesium sulfate (MERCK, KGaA), 0.05 g of manganese (II) sulfate (MERCK, KGaA), and 100 mM potassium phosphate buffer (MERCK, KGaA), and was prepared as previously described by Hugenschmidt et al. (2011) and modified by Chamlagain et al. (2016). The cultures were prepared from 15% glycerol stock stored at −80°C by streaking on a PPA agar plate (1.5% agar and pH 7.4) and incubation at 30°C in anaerobic jars (Anaerocult, Merck, Germany) for 4 days to activate the cells. Subsequently, 10 mL of PPA or WBM inoculated with *P. freudenreichii* cells were incubated at 30°C for 4 days. These pre-cultures were then diluted 100-fold in fresh PPA and WBM (45 mL each) and optical density was measured at 600 nm (OD_600_) every 4 h, until the growth ceased. Each culture experiment was conducted in triplicate. The generation times during exponential growth (G) were calculated from an equation G = Δt/3.3^*^(log(n_2_/n_1_)) where Δt is the time difference between two points for which doubling was recorded, n_1_ is the average OD_600_ value at the earlier time point and n_2_ is the average OD_600_ value at the later time point. The reported values are average of the calculations from three subsequent measurements.

### Cultures for metabolite analyses

For measuring the B12 vitamin, sugar and the levels of short chain fatty acids the *P. freudenreichii* strain was cultured in 230 mL in triplicate as follows: fresh PPA and WBM media inoculated with *P. freudenreichii* pre-cultures were incubated at 30°C under an anaerobic atmosphere (Anaerocult, Merck, Germany). Anaerobic atmosphere in the first 3 days of incubation is thought to enhance the initial formation of the corrin ring, the backbone of vitamin B12 (Martens et al., 2002), as the biosynthetic enzymes δ-aminolevulinic acid synthase (HemA) and the δ-aminolevulinic acid dehydratase (HemB) were shown to be strongly inhibited by aeration (Menon and Shemin, 1967). Then, at 72 h post inoculation (hpi) the bottles were opened to allow in air, thus enabling biosynthesis of DMBI. The cells were incubated for a further 96 h with shaking at 150 rpm. The cell samples were taken at the time points corresponding to the mid-exponential phase (t1: PPA, 24 hpi; WBM, 30 hpi), stationary phase (t2: 72 hpi for both media), and at 168 hpi (t3, for both media). The cells were harvested by centrifugation (3,000 × g) for 10 min at 4°C and washed once with ice-cold PBS pH 7.0 (Oxoid, Hamshire, England). Cell pellets and supernatants were stored at −20°C until used.

### HPLC analysis of metabolic profiles

Lactose and organic acids (lactic acid, propionic acid, acetic acid, pyruvic acid, and succinic acid) were determined as described earlier (Hugenschmidt et al., 2011; Chamlagain et al., 2016). The samples were analyzed on a Waters HPLC system (Waters, Milford, MA, USA) with UV detection (210 nm) for acids and refractive index (RI) detection (HP 1047A, HP, USA) for sugars. The mobile phase was 10 mM sulfuric acid run in an isocratic flow rate of 0.6 mL/min through an Aminex HPX-87H column (7.8 × 300 mm, 9 μm particles; Bio-Rad, USA) maintained at 40°C. Lactose and acids standards were used to create a calibration curve. Quantification was based on an external standard method using a multilevel (*n* = 7) calibration curve (0.8–40 μg) for each metabolite. Averages of three biological replicates, injected twice (40 μl) were reported. The molar ratio between propionic and acetic acids produced was calculated.

### UHPLC analysis of vitamin B12 and pseudovitamin

The cobalamin (vitamin B12) and its analog form (pseudovitamin B12) in cells was analyzed as previously described (Chamlagain et al., 2015; Deptula et al., 2015). Briefly, the wet-cell mass from equal volumes of cultures was weighed and suspended with 10 mL of extraction buffer (pH 4.5, 8.3 mM sodium hydroxide, and 20.7 mM acetic acid). Subsequently, 100 μL of 1% sodium cyanide was added in order to convert all the upper ligands of vitamin B12 and its analogs to the most stable cyano forms, preventing loss of the compounds during heat extraction (Kumar et al., 2010). The suspension was vortexed and then extracted in a boiling water bath. After 30 min of heat extraction the tubes were cooled in an ice-bath, followed by centrifugation (6,900 × g; 10 min). The residue was suspended in 5 mL of extraction buffer (pH 6.2) and centrifuged. Both supernatants were combined and the pH adjusted to 6.2 with the pH 6.2 buffer. Following the filtration of the extracts through filter paper (Ø 90 mm, VWR, Leuven, Belgium), the volume was adjusted to 25 mL with the pH 6.2 buffer and filtered into UPLC vials via syringe filter (0.2 μm, Pall, MI, USA) to analyze with the UHPLC.

The UHPLC analysis was run on Waters UPLC system (Waters, Milford, USA) with separation on a HSS T3 C18 column (2.1 × 100 mm) and UV detection by photo diode array (PDA) detector at 361 nm. The mobile phase was a gradient flow of MilliQ water and acetonitrile (0.32 mL/min), both acidified with 0.025% trifluoroacetic acid. The column temperature was set at 30°C and the sample vials were kept at 4°C. The samples were injected twice with 10 μL in each injection. The vitamin B12 in sample extracts was quantified with an external calibration curve obtained by injecting a set of cyanocobalamin standards with concentrations ranging from 0.015 to 0.75 ng/μL. Because pseudovitamin B12 is not available commercially, the concentration of the pseudovitamin B12 in the sample extracts was calculated by comparing its peak area with that of cyanocobalamin.

### Comparative proteome analyses of *P. freudenreichii*

Proteome changes of *P. freudenreichii* cultured in two different growth media and at two time points of growth (t1, t2) under anaerobic conditions at 30°C was studied using two-dimensional gel electrophoresis (2DE) combined with SYPRO Orange (Sigma-Aldrich, St. Louis, USA) staining. The fluorescent proteomes were analyzed and compared using the SameSpots (Totallabs, Newcastle, England) software, after which the same gels were stained with silver for localization of the proteins selected for in-gel tryptic digestion and LC-MS/MS identification.

### Two-dimensional gel electrophoresis (2DE)

*P. freudenreichii* 2067 cells were cultured in PPA and WBM in six biological replicates (45 mL each) and 10 mL cells samples were harvested at two time points corresponding to the exponential (t1: PPA, 24 hpi; WBM, 30 hpi) and stationary growth phases (t2: 72 hpi for both media) of growth. Cells were washed once with ice-cold 50 mM Tris-HCl (pH 8.0) and disrupted by beating with 0.1 mm glass beads (Sigma Aldrich, St. Louis, USA) using Fast-Prep™-24 (MP Biomedicals, Solon, OH, USA) with 3 lysis cycles of 30 s at 6.5 m/s, separated by 1-min cooling on ice. The samples were purified using the 2-D Clean-Up kit (GE Healthcare, Amersham, England) and the protein concentrations were determined using the 2D Quant kit (GE Healthcare, Amersham, England). An equal amount of protein (50 μg) from each sample was solubilized in UTCT buffer (7 M urea, 2 M thiourea, 4% CHAPS, 30 mM Trisma Base, 1% IPG buffer pH 4–7, 5 mM DTT). Solubilized proteins were separated using isoelectric focusing (IEF) (Ettan IPG phore, GE Healthcare, Amersham, England) with IPG strips (11 cm, pH 4–7; GE Healthcare, Amersham, England) rehydrated overnight in De-Streak solution (GE Healthcare, Amersham, England) supplemented with 1% IPG buffer pH 4–7 (GE Healthcare, Amersham, England). Samples were applied on to strips using loading cups placed at the anode end. IEF was conducted at 20°C with following parameters: 500 V for 500 Vh, linear ramping to 1,000 V for 800 Vh, linear ramping to 6,000 V for 8,800 Vh, hold at 6,000 V for 2,900 Vh. The current limit was set to 50 μA per strip. After IEF program completed, the strips were equilibrated in buffers containing 50 mM Tris-HCl at pH 6.8, 6 M urea, 2% SDS, 20% glycerol, and alternatively either 2% DTT or 2.5% iodoacetamide (15 min each) and were loaded on 12.5% Tris-HCl Criterion Precast Gels (BioRad, Hemel Hempstead, England). The second dimension separation of proteins was conducted using the Criterion Dodeca Cell (BioRad, Hemel Hempstead, England) in Tris-Glycine-SDS buffer (BioRad, Hemel Hempstead, England).

After electrophoresis, the gels were stained with SYPRO Orange dye (Sigma-Aldrich, St. Louis, USA; 1:5,000) using the previously described protocol (Malone et al., 2001) with the following modifications: the gels were fixed in a solution composed of 40% ethanol, 2% acetic acid, 0.0005% SDS for 1 h, and the pre-stain washing step was omitted while two post-staining rinses with MilliQ water were used to remove excess stain from the gels. After the last rinsing step, the fluorescent gel images were directly captured using the AlphaImagerHP documentation system (ProteinSimple, Santa Clara, California, USA). 2DE images were analyzed using the SameSpots software v4.5 (Totallab, Newcastle, England). Non-spot data from the 2DE images were first removed by cropping the images, which was followed by automatic normalization and selection of the reference image. Spot matching between different 2DE proteomes was manually improved by adding appropriate number of alignment vectors to each gel image. Spot volumes for all matched proteins were calculated and normalized to the total spot volume for calculating average spot volume changes across all replicates within both test groups. Principal component analysis was used to compare the effect of the growth condition and growth stage on the proteome of the studied strain. Finally, spots showing fold-changes ≥1.4–1.5 between the two conditions (PPA vs. WBM) and with ANOVA *p* < 0.05 were picked for in-gel tryptic digestion and LC-MS/MS analysis.

### Protein identification

The fluorescent 2DE gels were re-stained with silver nitrate using the mass-spectrometry compatible method described by O'Connell and Stults (1997) with following modification; the concentration of acetic acid was reduced to 0.5% in the fixing step. Spots that met the selection criteria were subjected to trypsin digestion (Shevchenko et al., 1996) followed by LC-MS/MS identification (Ultimate 3,000 nano-LC, Dionex, Hvidovre, Denmark) and QSTAR Elite hybrid quadrupole TOF mass spectrometry (Applied Biosystems/MDS Sciex with nano-ESI ionization, Sciex Carlsbad, California, USA) as previously described (Öhman et al., 2010).

The LC-MS/MS data were searched using the Mascot search algorithm (Matrix Science, version 2.2.03, London, UK) against the NCBI database with taxonomy set to “Other Actinobacteria (class)” through the ProteinPilotTM3.0 interface (version 2.0.1, Applied Biosystems/MDS SCIEX, Carlsbad, California, USA). The search criteria for Mascot searches were: trypsin digestion with one missed cleavage allowed, carbamidomethyl modification of cysteine as a fixed modification, and oxidation of methionine, phosphorylation of serine, threonine, and tyrosine as variable modifications. For the LC-MS/MS spectra, both the maximum precursor ion mass tolerance and MS/MS fragment ion mass tolerance were 50 ppm, and a peptide charge state of +1, +2, +3 was used. A successful identification was reported when a significant match (*p* < 0.05) with at least two peptide hits (ion score > 40) was obtained.

## Results

### Growth kinetics

**Figure 1:**
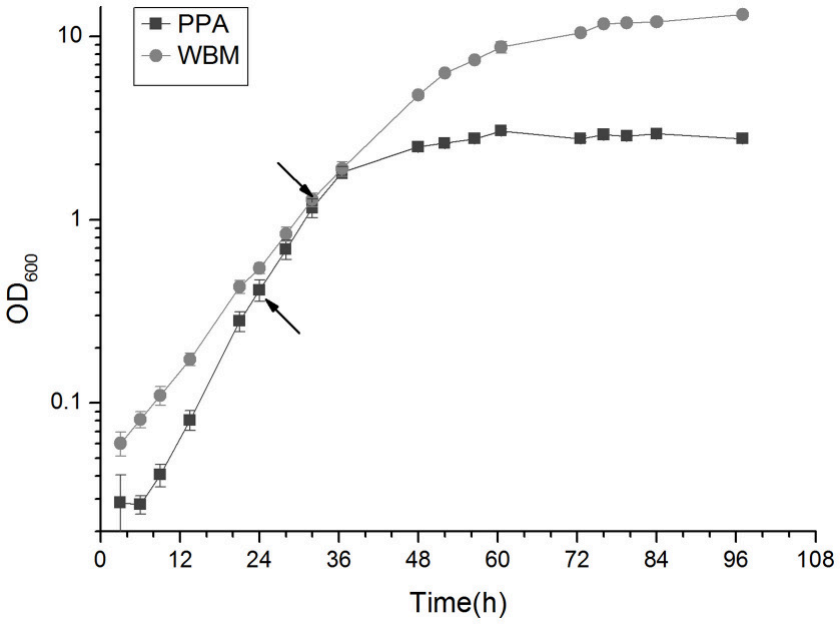
**Growth curve of ***P. freudenreichii*** in PPA and WBM under anaerobic conditions at 30°C**. Exponential sampling time points (t1) at 24 h for PPA and 30 h for WBM calculated from the growth curve are marked with arrows.

The generation times of the *P. freudenreichii* 2067 strain in PPA and WBM under anaerobic conditions were 4.2 h (SD, 0.0) and 5.8 h (SD, 0.2), respectively. In PPA, the cell density measured at the end of the growth (72 h) was 2.8 (SD, 0.1), while in WBM a value of 10.5 (SD, 0.3) was reached (Figure 1). The growth curve in Figure 1 indicates that the mid- exponential growth phase was reached at 24 and 30 hpi in PPA and WBM, respectively. These growth stages were used as the first sampling time points (t1) in the following experiments.

### Metabolic activity of *P. freudenreichii* in PPA and WBM

The metabolites of the selected *P. freudenreichii* strain were assessed from cell samples harvested at three time points, corresponding to the mid-exponential t1 (24/30 hpi), stationary t2 (72 hpi) and late stationary t3 (168 hpi) growth phases at 30°C. Here, a two-step incubation method previously used to maximize B12 vitamin yield by *P. freudenreichii* (Marwaha et al., 1983; Martens et al., 2002; Hugenschmidt et al., 2011) was followed. For this purpose, the cultures were first grown under anaerobic conditions and at 72 hpi the cells were exposed to mild aeration at 30°C until 168 hpi was reached.

#### Vitamin B12

**Figure 2:**
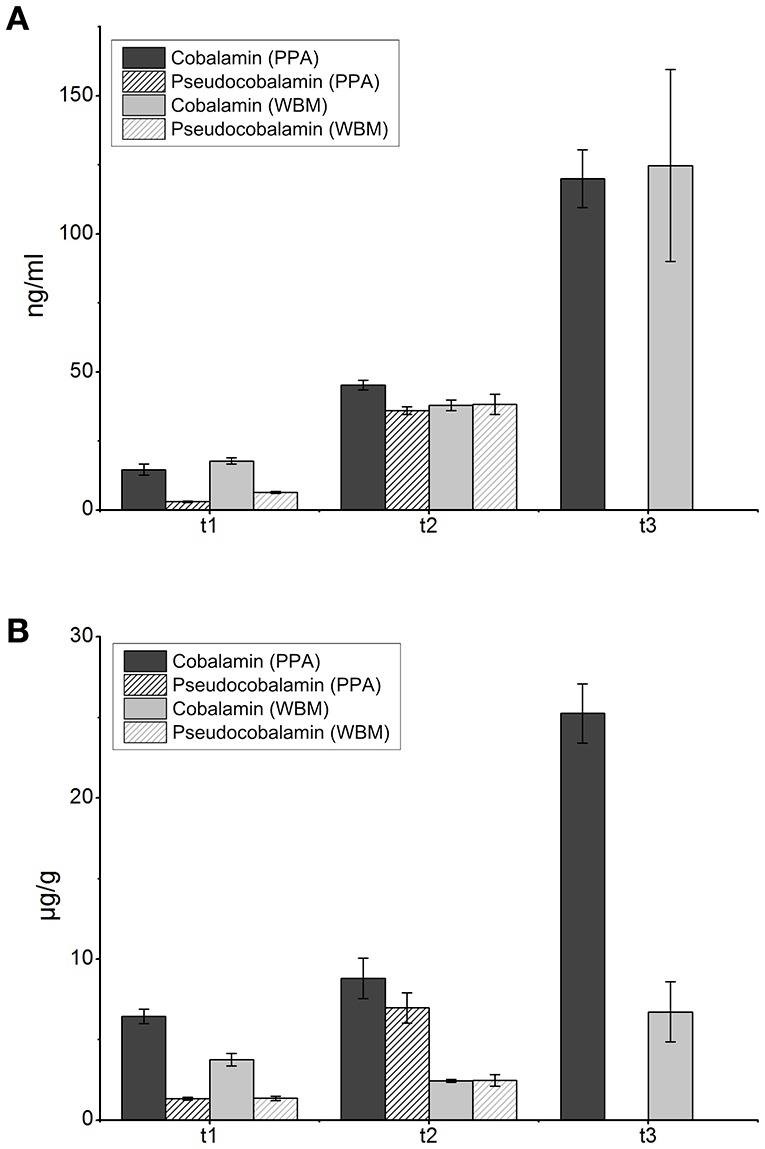
**Levels of cobalamin (full) and pseudocobalamin (cross-hatched) measured from cells cultured in PPA (dark) and WBM (light) at the indicated three time points: t1- mid-exponential growth phase (24 h for the PPA-grown cultures, 30 h for WBM-grown cultures as determined by the growth curve); t2- stationary phase at 72 h and t3- late stationary phase at 168 h. (A)**, Cobalamin and pseudocobalamin levels expressed in ng per ml of culture **(B)**, cobalamin and pseudocobalamin levels expressed in μg per g wet cells. Values are an average of three biological replicates.

From exponentially growing cells (harvested at t1) the levels of cobalamin (<20 ng/ml) and pseudocobalamin (<7 ng/ml) were similar in cells grown in both media (Figure 2A). At the onset of stationary phase (t2), slightly higher levels of cobalamin (45.3 ± 1.7 ng/ml) were detected from cells grown in PPA than in to those from WBM (37.9 ± 1.9 ng/ml), and the levels of pseudocobalamin remained similar in both cultures (below 40 ng/ml; Figure 2A). After the time point t3 cobalamin levels of 124.8 ± 34.7 and 120 ± 10.4 ng/ml were detected in the cells grown in WBM and PPA, respectively (Figure 2A). Notably, the level of pseudocobalamin was below the detection limit in samples withdrawn at this time point. For the purpose of relative comparison, the amount of cobalamin produced was recalculated as per gram of wet cells, and was found to be higher in PPA grown cells than in WBM grown cells in each sampling time point (Figure 2B).

#### Sugars and acids

**Figure 3:**
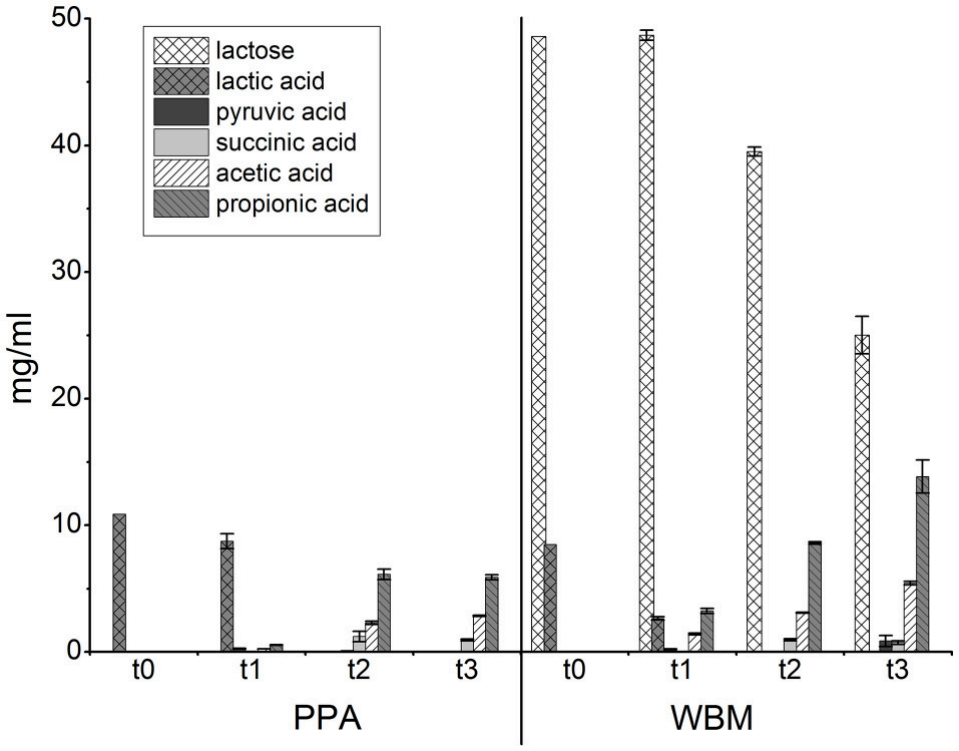
**Levels of the indicated metabolites in the PPA and WBM culture supernatants of ***P. freudenreichii*****. t0 refers to uncultured media that were used as the negative control in the experiments; t1—24 h in PPA, 30 h in WBM; t2—72 h; t3—168 h.

The amounts of lactose, glucose, lactic acid, succinic acid, pyruvic acid, propionic acid, and acetic acid were followed during growth (Figure 3). Lactic acid was present in both media (10.9 mg/ml in PPA; 8.5 mg/ml in WBM) and was apparently consumed already in the initial growth phases, as evidenced by its reduced levels in samples at t1 from both media. Notably, in WBM lactic acid was mostly consumed already at the exponential growth phase. In both of the media the level of lactic acid was below the detection limit in the samples harvested at stationary phase (t2). PPA contained no lactose whereas the initial level of lactose in WBM (48.6 mg/ml; t0) remained unchanged during exponential growth phase (t1), but was consumed during stationary phase (t2), and at the end of incubation (t3) to a level of 25 ± 1.5 mg/ml. The low levels of propionic and acetic acids produced in the exponential phase (t1) in PPA was proportional to the consumption of lactic acid and subsequently increased in stationary phase (t2) to 6.1 ± 0.4 and 2.3 ± 0.1 mg/ml, to reach final (t3) concentrations of 5.9 ± 0.2 and 2.9 ± 0.0 mg/ml, respectively. The final (t3) concentration of propionic and acetic acid at 13.8 ± 1.3 and 5.4 ± 0.1 mg/ml, respectively, was considerably higher in WBM, and accompanied by a pH drop from 6.4 to 4.6. The initial (t0) pH of the PPA medium (6.0) increased to approximately 6.2 at 72 hpi and remained stable until the end of cultivation. The calculation of molar ratio of propionic acid to acetic acid revealed that during exponential growth (t1) the ratio was comparable for both media at the levels of 1.9 ± 0.1 for PPA and 1.9 ± 0.1 for WBM. The proportions started shifting during stationary phase (t2) to 2.2 ± 0.0 and 2.3 ± 0.0 to finally reach 1.8 ± 0.1 and 2.1 ± 0.1 at 168 hpi (t3) for PPA and WBM, respectively. Small amount of pyruvic acid was detected in the exponential growth phase (t1) in PPA culture, but were found depleted in the stationary phase (t2). In WBM, pyruvic acid was detected in the samples from exponential phase as well as in the final sample (168 hpi), but not in the stationary phase sample harvested at 72 hpi. Finally, succinic acid was detected in both media at stationary phase (t2) samples, with the levels (1.2 ± 0.4 mg/ml in PPA; 1.0 ± 0.1 mg/ml in WBM) slightly decreasing toward the end of the incubation.

### Proteome changes of *P. freudenreichii* cultured in PPA and WBM

**Figure 4:**
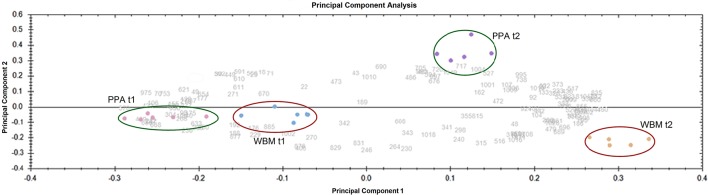
**Principal Component Analysis (PCA) of the gel images obtained from all conditions tested**. Biological replicates are circled.

**Figure 5:**
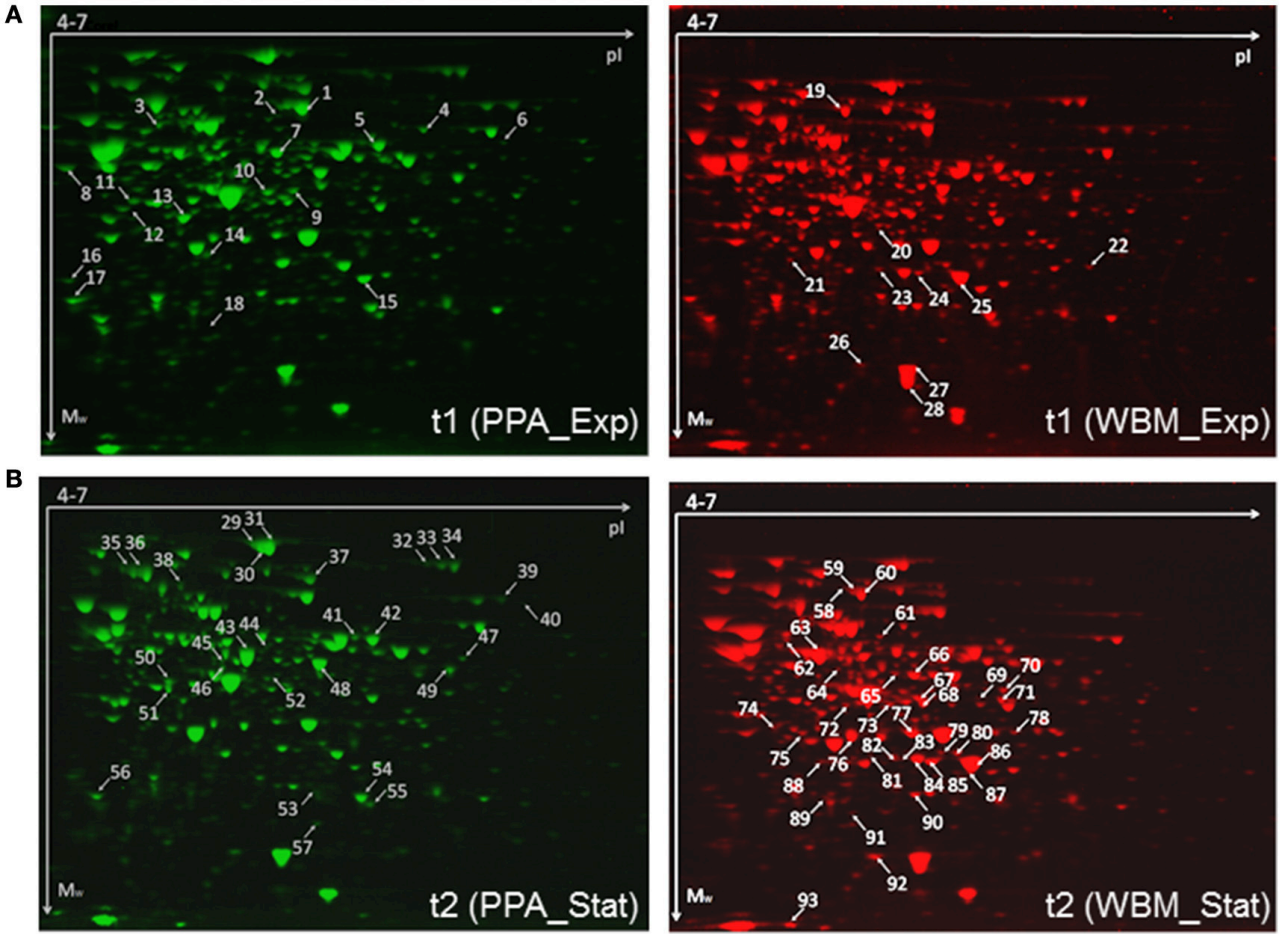
**Representative exponential (A)** and stationary **(B)** phase associated proteomes of the *P. freudenreichii* cells cultured in PPA (left) and WBM (right). Spots with relative abundance fold-change ≥1.4 (ANOVA, *p* ≤ 0.05) are marked with arrows and numbers. Raw gel-images are provided as Supplementary Material (Figures S1–S4).

Cells were harvested from PPA and WBM cultures at two time points (t1 and t2) and proteins extracted and purified from each sample were subjected to 2DE analyses combined with Sypro Orange staining to produce fluorescent proteomes. The 2DE images analyzed by SameSpots software enabled detection of approximately 600 and more than 700 protein spots from exponential and stationary growth phase associated samples, respectively. Principal Component Analysis (PCA) was next applied to visualize the differences in the protein abundance profiles between the PPA and WBM associated proteomes (Figure 4). The PCA, while demonstrating high reproducibility between the independent replicate samples, reveals that the exponential stage samples cluster closer together than those from the stationary phase. Thus, PCA results clearly indicated that the protein expression profiles are more alike during exponential growth in PPA and WBM, and that stationary phase induces more proteome changes in the studied strain. Figure 5 shows representative fluorescent proteomes in the pI range of 4–7 of the indicated strain at exponential (Figure 5A) and stationary (Figure 5B) stages of growth.

According to SameSpots analyses, 22 protein spots showed 1.5-fold change in spot volume values (*p* ≤ 0.05) in exponential stage (t1) proteomes (Figure 5A), whereas the number of protein spots showing changed abundances was 65 in the stationary phase (t2) associated proteomes (Figure 5B). As significantly more protein spots were found to be differentially expressed during the stationary phase, we decided to test if the number of the protein spots showing statistically significant change could be increased after applying less-stringent quantification criteria on the exponential phase proteomes. Reducing the fold-change criteria down to 1.4-fold increased the number of changed protein spots up to 28; 18 spots with fold-change ≥ 1.4 and *p* < 0.05 (fifteen spots with fold-change ≥ 1.5, *p* < 0.05) were more abundant in the PPA-associated proteome (Figure 5A), whereas 10 spots were exclusively more abundant in the WBM proteome (Figure 5B). In the case of the stationary phase samples, 29 of the spots were found to be more abundant in the PPA proteome and 36 in the WBM proteome (fold-change ≥ 1.5, *p* < 0.05). Proteins identified by LC-MS/MS from each spot are listed in Tables S1, S2. In total, 51 and 112 gene products were identified from the exponential and the stationary phase proteomes, respectively. From these identifications, only 10 (exponential) and 21 (stationary phase) with fold-change ≥ 1.5 (*p* < 0.05) were exclusively identified from one single protein spot (Tables 1, 2).

**Table 1:** **List of proteins detected by LC-MS/MS from spots with relative abundance fold change ≥ 1.5 (ANOVA, ***p*** ≤ 0.05) from PPA and WBM media during exponential growth phase**.

**Spot no.^a^**	**Fold change**	**Mw^b^**	**pI^b^**	**Mascot score**	**No of match.pept.^c^**	**Seq. cov.^c^(%)**	**Locus in reference organism^d^**	**Name**	**Annotation**
**PPA**									
2	1.5	64546	5.2	709	9	22	PFREUD_11940	GlnRS	Glutaminyl-tRNA synthetase
6	1.5	56027	5.8	274	2	20	PFREUD_13090	MiaB1	2-methylthioadenine synthetase
8	2.2	52579	4.7	1706	30	69	PFREUD_10490	AtpD	ATP synthase subunit beta
14	1.5	31894	4.8	487	9	15	PFREUD_09430	FabH	3-oxoacyl-ACP synthase
16	2.3	29633	4.5	676	12	51	PFREUD_02490	FixB	Electron transfer flavoprotein FixB
18	1.6	23335	5.0	530	7	44	PFREUD_23970	SDR	Oxidoreductase
**WBM**									
24	1.5	33620	5.1	903	16	58	PFREUD_16420	Cys2	Cysteine synthase A
25	1.9	33620	5.1	1557	34	82	PFREUD_16420	Cys2	Cysteine synthase A
26	1.5	22619	5.3	392	4	26	PFREUD_06110	SodA	Superoxide dismutase
27	1.5	22619	5.3	611	16	47	PFREUD_06110	SodA	Superoxide dismutase

*Only spots with single identification are presented*.

a *Spot numbers refer to those shown in Figure 5A*.

b *Theoretical molecular weight (Mw) and pI values were obtained*.

c *No. match. pept., number of matched peptides; Seq. cov., sequence coverage*.

d *P.freudenreichii CIRM-BIA1^T^ GenBank: FN806773.1*.

**Table 2:** **List of proteins detected by nano-LC-MS/MS from spots with relative abundance fold change ≥ 1.5 (ANOVA, ***p*** ≤ 0.05) from PPA and WBM media during stationary growth phase**.

**Spot no.^a^**	**Fold change**	**Mw^b^**	**pI^b^**	**LC-MS/MS score**	**No of match. pept.^c^**	**Seq. cov. (%)^c^**	**Locus in reference organism^d^**	**Name**	**Annotation**
**PPA**									
29	1.6	137,778	5.2	2689	40	33	PFREUD_01840	NifJ1	Pyruvate synthase/pyruvate-flavodoxin oxidoreductase
30	1.9	137778	5.2	2525	59	34	PFREUD_01840	NifJ1	Pyruvate synthase/pyruvate-flavodoxin oxidoreductase
31	1.7	137,778	5.2	3124	71	42	PFREUD_01840	NifJ1	Pyruvate synthase/pyruvate-flavodoxin oxidoreductase
36	1.7	96,275	4.8	2351	50	39	PFREUD_03230	PPDK	Pyruvate phosphate dikinase
37	2.3	94,317	5.3	2756	48	44	PFREUD_17920	ClpB2	ATP-dependent Clp protease B2
39	2.4	75,276	6.0	1488	36	36	PFREUD_14311	SdhA3	Succinate dehydrogenase flavoprotein subunit
42	1.9	60,757	5.5	1966	67	62	PFREUD_01830	GLS	FAD-dependent pyridine nucleotide-disulfide oxidoreductase
43^e^	3.6	53,385	5.1	825	62	25	PFREUD_16320	AspA1	Aspartate ammonia-lyase
43^e^	3.6	53,526	5.1	748	48	26	PFREUD_16330	AspA2	Aspartate ammonia-lyase
46	2	47,518	5.0	270	4	11	PFREUD_01350	SerRS1	Seryl-tRNA synthetase
47	1.5	47,953	5.7	678	11	27	PFREUD_05140	GGR	Electron transfer oxidoreductase (Geranylgeranyl reductase superfamily)
57	1.6	24,018	5.2	352	21	33	PFREUD_22440	SDR_c	Short subunit dehydrogenase (classical)
**WBM**									
59	2	93,458	5.1	2766	46	50	PFREUD_19250	ClpB1	ATP-dependent Clp protease B1
60	1.5	93,458	5.1	2527	49	46	PFREUD_19250	ClpB1	ATP-dependent Clp protease B1
70	2	49,226	5.5	1290	20	51	PFREUD_17610	GDH	glutamate dehydrogenase
75	3	34,225	4.9	661	15	31	PFREUD_12840	L-LDH	L-lactate dehydrogenase
78	1.6	35,532	5.2	609	8	27	PFREUD_15130	GAPDH	Glyceraldehyde-3-phosphate dehydrogenase
81	2	32,368	5.0	582	8	34	PFREUD_23890	FBA2	Fructose-bisphosphate aldolase class I
85	3.7	33,620	5.1	431	6	28	PFREUD_16420	Cys2	Cysteine synthase A
86	2.2	33,620	5.1	826	47	54	PFREUD_16420	Cys2	Cysteine synthase A
91	1.8	23,335	5.0	476	6	46	PFREUD_23970	SDR	Oxidoreductase
92	1.5	22,619	5.3	314	6	18	PFREUD_06110	SodA	Superoxide dismutase
93	5.2	16,830	4.9	575	8	58	PFREUD_09500	IbpA	Molecular chaperone (small heat shock protein)

*Only spots with single identification are presented*.

a *Spot numbers refer to those shown in Figure 5A*.

b *Theoretical molecular weight (Mw) and pI values were obtained*.

c *No. match. pept., number of matched peptides; Seq. cov., sequence coverage*.

d *P.freudenreichii CIRM-BIA1^T^ GenBank: FN806773.1*.

e *In the spot 43 two proteins were identified; both were isoforms of aspartate ammonia-lyase and are therefore included in the table*.

Proteins that were more produced during the exponential growth phase in PPA included lipid metabolism (3-oxoacyl-ACP synthase—FabH), energy production (ATP synthase subunit beta—AtpD, electron transfer flavoprotein—FixB), and protein production (glutaminyl-tRNA synthetase—GlnRS, 2-methylthioadenine synthetase—MiaB1) associated proteins (Table 1). Two potential oxidoreductases (SDR and FAD-dependent pyridine nucleotide-disulfide oxidoreductase) were also among the most abundant proteins in *P. freudenreichii* during exponential growth in PPA. Proteins identified as more abundant from the WBM proteome included oxidative-stress associated cysteine synthase 2 (Cys2) and a superoxide dismutase (SodA) identified from two separate spots (Table 1).

More abundant proteins associated with the stationary phase (t2) proteomes of the PPA grown cells were identified as proteins involved in energy generation through metabolism of pyruvate (NifJ1, PPDK) or feeding the TCA cycle (SdhA3, AspA1/2, and GLS). Stress response related ClpATPase (ClpB2), protein synthesis associated SerRS1 and a potential dehydrogenase (SDR_c) were identified at elevated levels from PPA cultured cells at stationary phase. In WBM grown cells, proteins involved in glycolysis/utilization of lactic acid (L-LDH, GAPDH, FBA2), nitrogen metabolism (GDH), and stress (SDR, ClpB1, Cys2, SodA, IbpA) were all identified at elevated levels (Table 2).

## Discussion

The analyses of growth kinetics of *P. freudenreichii* 2067 in PPA and WBM media revealed notable differences including a 1.5-h shorter generation time in PPA during the exponential growth, while a nearly 4-times greater cell density value at 72 hpi was observed in WBM. Faster growth rates in PPA suggests that conditions are more favorable in this medium, whereas the higher abundance of carbon sources and nutrients in WBM allow growth to higher cell-densities after longer incubation periods.

According to previous studies, *P. freudenreichii* preferentially uses lactic acid in the presence of multiple carbon sources (Piveteau, 1999). Consistent with this finding, we observed no lactose consumption until lactic acid was depleted from the WBM medium. Small amounts of pyruvic acid were detected in both media at the exponential growth phase, while in the stationary phase its level was below detection limits. Excretion of pyruvic acid to maintain its cellular levels below toxic level is typical under conditions rich in lactic acid, followed by its intake and reuse after depletion of lactic acid (Crow, 1986a; Dalmasso et al., 2012a). When grown on lactate medium, *P. freudenreichii* typically produces propionic and acetic acids in molar ratio of 2:1 (Piveteau, 1999; Crow, 1986b). The lower ratio for the cells grown in PPA of propionic to acetic acid than was expected may be due to exhaustion of lactic and pyruvic acids, directing the cells to switch to amino acid metabolism, which in turn results in an increased amount of acetic acid (Crow, 1986b). The presence of unused lactose at the 168 hpi in WBM suggests that the growth in this medium was inhibited by low pH rather than exhaustion of carbon sources, as was the case in PPA.

In *P. freudenreichii*, cobalamin is produced mainly at the later stages of growth (Bullerman and Berry, 1966). In agreement with that, we observed the highest increase in levels of cobalamin between 3 and 7 days of incubation, although at relatively low levels of ~120 ng/ml. In our previous study, cobalt chloride supplementation (5 mg/l) allowed the same *P. freudenreichii* strain to produce nearly 2.5 μg/ml of cobalamin in WBM (Chamlagain et al., 2016). Considering that the amount of cobalamin produced in the present study is typical for cultures grown without cobalt supplementation (Hugenschmidt et al., 2011), it can be assumed that cobalt was a limiting factor in cobalamin production here. According to a U.S. patent (Hargrove and Abraham, 1955), the higher growth of *P. freudenreichii* is generally associated with higher production of B12, assuming that surplus of cobalt is available. However, addition of cobalt is not desirable from the perspective of food fortification. Taking into account a newly updated adequate intake (AI) of cobalamin set at 4 μg per day for adults (EFSA NDA Panel, 2015), consumption of 34 ml of product fermented with *P. freudenreichii* 2067 producing cobalamin at the level of 120 ng/ml would meet nutritional requirements, without the need for the addition of cobalt. In future approaches, matrices naturally rich in cobalt could be selected to increase cobalamin production.

Interestingly, small amounts of pseudocobalamin were observed in both media at the exponential and stationary growth phases. Notably, the level of pseudovitamin was below the detection limit at the end of incubation, indicating conversion of the pseudovitamin to active vitamin. The mechanism behind the conversion remains to be elucidated for *P. freudenreichii*, however, replacement of lower ligand for the one preferred by the bacterium was previously described for *Dehalococcoides mccartyi* (Gray and Escalante-Semerena, 2009; Yi et al., 2012) and also observed in other species (Allen and Stabler, 2008; Keller et al., 2014). This initial production of pseudovitamin could be due to oxygen limitation during the first 72 h of incubation, since it was recently shown that DMBI, the lower ligand of the active vitamin B12, cannot be made under strictly anaerobic conditions (Deptula et al., 2015). Introduction of oxygen during the later stages of experiment allowed for synthesis of DMBI, which is the purpose of employing the two-step incubation (Martens et al., 2002). Therefore, oxygen accessibility during the fermentation process should be considered in food applications aiming to achieve B12 fortification and avoid the production of pseudovitamin.

Multivariate analysis of the fluorescent 2DE-proteomes indicated greatest differences in stationary phase associated samples, and that the compositional differences of the tested growth media had only marginal effect on the exponentially growing cells. Thus, it can be assumed that the proteins identified from the differentially abundant spots reflect the differing composition of the two media used. For example, the PPA medium contains tryptone, yeast extract and the sodium lactate, while WBM contained Tween 80 as a source of lipids and whey permeate that is rich in nutrients such as the carbon source in the form of lactose. Cells growing in PPA without lipid sources have elevated levels of proteins such as FabH and MaoC, which are involved in the lipid metabolism. The higher abundance of enzymes involved in protein synthesis (MiaB1 and GlnRS) in the proteomes of cells grown in PPA is in accordance with the higher growth rate in this medium. The relatively low final cell density measured for the strain in PPA medium (OD_600_ ~ 2.8) with a relatively high pH (pH = 6.2) can be attributed to the depletion of energy sources rather than inhibition resulting from production of acids, since only one stress-associated protein (ClpB2) was detected as more abundant protein under these conditions. Conversely, growth of the cells in WBM resulted in considerably higher final cell densities (OD_600_ ~10.5) with the pH-value of the culture dropping to ~4.5, which has been shown to inhibit the growth of non-adapted *Propionibacterium* cells (Rehberger and Glatz, 1998). The increased abundance of several stress-related proteins (ClpB1, Cys2, and SodA) in WBM grown cells indicates activation of stress response mechanisms under these conditions in *P. freudenreichii*, which is likely to improve viability and adaptation of the strain in whey-based medium. The switch to lactose utilization at the later growth stages in WBM is reflected by the increased abundance of different glycolytic proteins such as GAPDH and FBA2 (Table 2). On the other hand, SdhA3 that is an enzyme involved in Wood-Werkman and TCA cycles was identified from a more abundant spot from the PPA proteome. The metabolism of pyruvate in PPA at the stationary phase could explain the increased abundance of Nifj1 and PPDK (Table 2). Furthermore, the increased production of two aspartate ammonia lyases during stationary-phase in PPA may indicate that *P. freudenreichii* employs amino acid metabolism for energy generation in this medium in the face of carbon source limitation, which is in agreement with the higher proportion of acetic acid produced (Crow, 1986b).

Five out of over 20 vitamin B12 biosynthetic proteins were identified from spots differentially abundant in PPA and WBM proteomes (Data Sheet 1, Table S1), including delta-aminolevulinic acid dehydratase (HemB) at the exponential growth phase and then both isoforms of glutamate-1-semialdehyde 2,1-aminomutase (HemL1 and HemL2), precorrin-2 C20-methyltransferase (CbiL), and the ATP-binding protein of the cobalt transporter (CbiO) at stationary phase (Data Sheet 1, Table S2). However, from all of these spots multiple protein identifications were obtained and no cobalamin-biosynthetic proteins were detected as neither single nor the best hit. Therefore, it can be assumed that, under the conditions tested, the abundance of B12 biosynthesis proteins did not significantly differ between PPA and WBM cultures.

In conclusion, growth of *P. freudenreichii* in the two media differed markedly, with a considerably higher final cell density and higher yield of propionic and acetic acids in WBM, concurrent with the acidification of the medium and increased production of stress proteins. However, the total production of the vitamin B12 was almost equal in both media, which was also reflected in the absence of obvious differences in the abundances of B12 biosynthetic proteins. Consequently, the lower final cell density in PPA coincided with considerably higher intra-cellular accumulation of vitamin B12. In environments where cobalt is limited, such as food matrices and the growth media without added cobalt, the production appears to be independent of growth and metabolic state of the cells. A high intracellular accumulation of vitamin B12 combined with high growth yields would be desirable from the perspective of industrial production of cobalamin for use as vitamin supplements or bacterial supplements. However, in the context of fortification of foods, the production levels observed in this study would require consumption of only small amounts of product fermented with *P. freudenreichii* to meet the recommended daily vitamin B12 consumption levels. Furthermore, the results suggest that levels of short chain fatty acids: propionic and acetic acids could be modulated by the availability of a usable carbon source, depending on the desirability of these acids in the final products.

## Author contributions

PV, VP, and KS conceived the study. PD, BC, PS, ME, and TN acquired the data. All authors contributed to the interpretation of the work, wrote, and approved the manuscript and agree to be accountable for all aspects of the work.

## Funding

This work was supported by the Academy of Finland [grant numbers 257333 and 272363].

### Conflict of interest statement

The authors declare that the research was conducted in the absence of any commercial or financial relationships that could be construed as a potential conflict of interest.

## Abbreviations

DMBI: 5,6-dimethylbenzimidazole
PPA: cheese-like propionic medium
WBM: industrial-type whey-based medium.
