# Supplementary material for: Food-Like Growth Conditions Support Production of Active Vitamin B12 by Propionibacterium freudenreichii 2067 without DMBI, the Lower Ligand Base, or Cobalt Supplementation
Source: Front Microbiol. 2017 Mar 8;8:368. doi: 10.3389/fmicb.2017.00368 (PMC5340759; doi:10.3389/fmicb.2017.00368)
Supplement: Supplementary file 1 [file DataSheet1.pdf]

Supplementary material

Food-like growth conditions support production of active vitamin B12 by *Propionibacterium freudenreichii* without DMBI or cobalt supplementation.

P. Deptula, B. Chamlagain, M. Edelmann, P.Sangsuwan, T. A. Nyman, K. Savijoki, V. Piironen, P. Varmanen

*** Correspondence:** Pekka Varmanen pekka.varmanen@helsinki.fi

16S rDNA sequence analysis

The *P. freudenreichii* strain 2067kk (Valio Ltd) isolated from dairy environment was subjected to genotypic analysis by the partial sequencing of a 500 bp internal fragment (nucleotides 27–518) of the 16S rDNA gene. The strain was cultured in 10 mL of PPA medium at 30°C for four days under microaerophilic conditions. Cells were harvested by centrifugation (3220 g, 10 min, 4°C) and washed once with ice-cold 50mM TRIS pH 8.0. Cells were disrupted with glass beads (≤106 µm in diameter; Sigma) in a Fast Prep-24 homogeniser (MP Biomedicals, France) in three 30-seconds 6,5 m/s cycles with one-minute cooling on ice between the cycles. PCR was performed from cell lysates using the universal 16S rDNA primers p8F (5′AGAGTTTGATCCTGGCTCAG3′) and pfRev (5′ACGAGCTGACGACAGCCATG3′) (Edwards et al., 1989), and the PCR products were sequenced using a BigDye terminator cycle sequencing kit with the primer pdRev (5′GTATTACCGCGGCTGCTG3’), in the DNA sequencing laboratory of the Institute of Biotechnology, University of Helsinki, Helsinki, Finland. Sequencing reactions were run on an ABI 3700 automated DNA Sequencer (Applied Biosystems, Foster City, CA, USA). The sequence data was compared to all 16S-rRNA sequences of bacteria in the gene bank, using the BlastN tool (http://www.ncbi.nlm.nih.gov /BLAST/). The Blast search using the obtained sequence as query revealed 100% identity with the annotated *Propionibacterium freudenreichii* subsp. *shermanii* CIRM-BIA (Figure S1) 16S rDNA gene sequence (Falentin et al. 2010), while 99% identity to the sequence from the type strain of *Propionibacterium freudenreichii* subsp. *freudenreichii* DSM 20271 (Koskinen et al. 2015) was found and therefore we concluded that the identity of the selected strain is *P. freudenreichii* subsp. *shermanii*.

**Score**	**Expect**	**Identities**	**Gaps**	**Strand**
1421 bits(769)	0.0	769/769(100%)	0/769(0%)	Plus/Plus

```
Query  1        CCCCGTGGAAGGGACCCACACCTAGTACCCACCGTTTACGGCGTGGACTACCAGGGTATC  60
                ||||||||||||||||||||||||||||||||||||||||||||||||||||||||||||
Sbjct  1539504  CCCCGTGGAAGGGACCCACACCTAGTACCCACCGTTTACGGCGTGGACTACCAGGGTATC  1539563

Query  61       TAAGCCTGTTTGCTCCCCACGCTTTCGCTTCTCAGCGTCAGGAAATGTCCAGAGAACCGC  120
                ||||||||||||||||||||||||||||||||||||||||||||||||||||||||||||
Sbjct  1539564  TAAGCCTGTTTGCTCCCCACGCTTTCGCTTCTCAGCGTCAGGAAATGTCCAGAGAACCGC  1539623

Query  121      CTTCGCCACTGGTGTTCTTCCCGATATCTGCGCATTCCACCGCTCCACCGAGAGTTCCAT  180
                ||||||||||||||||||||||||||||||||||||||||||||||||||||||||||||
Sbjct  1539624  CTTCGCCACTGGTGTTCTTCCCGATATCTGCGCATTCCACCGCTCCACCGAGAGTTCCAT  1539683

Query  181      TCTCCCCTACATTCCTCAAGTCAACCCGTATCGAAAGCAAGCCCAGAGTTAAGCCCTGGA  240
                ||||||||||||||||||||||||||||||||||||||||||||||||||||||||||||
Sbjct  1539684  TCTCCCCTACATTCCTCAAGTCAACCCGTATCGAAAGCAAGCCCAGAGTTAAGCCCTGGA  1539743

Query  241      ATTTCACTTCCGACGTGATCAACCGCCTACAAGCTCTTTACGCCCAATAATTCCGGACAA  300
                ||||||||||||||||||||||||||||||||||||||||||||||||||||||||||||
Sbjct  1539744  ATTTCACTTCCGACGTGATCAACCGCCTACAAGCTCTTTACGCCCAATAATTCCGGACAA  1539803

Query  301      CGCTCGCACCCTACGTATCACCGCGGCTGCTGGCACGTAGTTAGCCGGTGCTTCTTCTCC  360
                ||||||||||||||||||||||||||||||||||||||||||||||||||||||||||||
Sbjct  1539804  CGCTCGCACCCTACGTATCACCGCGGCTGCTGGCACGTAGTTAGCCGGTGCTTCTTCTCC  1539863

Query  361      CACTACCGTCACTTGCGCTTCGTCATGGATGAAAGCGGTTTACAACCCGAAGGCCGTCAT  420
                ||||||||||||||||||||||||||||||||||||||||||||||||||||||||||||
Sbjct  1539864  CACTACCGTCACTTGCGCTTCGTCATGGATGAAAGCGGTTTACAACCCGAAGGCCGTCAT  1539923

Query  421      CCCGCACGCGGCGTTGCTGCATCAGGCTTGCGCCCATTGTGCAATATTCCCCACTGCTGC  480
                ||||||||||||||||||||||||||||||||||||||||||||||||||||||||||||
Sbjct  1539924  CCCGCACGCGGCGTTGCTGCATCAGGCTTGCGCCCATTGTGCAATATTCCCCACTGCTGC  1539983

Query  481      CTCCCGTAGGAGTCTGGGCCGTATCTCAGTCCCAATGTGGCCGGTCACCCTCTCAGGCCG  540
                ||||||||||||||||||||||||||||||||||||||||||||||||||||||||||||
Sbjct  1539984  CTCCCGTAGGAGTCTGGGCCGTATCTCAGTCCCAATGTGGCCGGTCACCCTCTCAGGCCG  1540043

Query  541      GCTACCCGTCGCTGCCTTGGTAGGCCATTACCCCACCAACAAGCTGATAGGCCGCGAGAC  600
                ||||||||||||||||||||||||||||||||||||||||||||||||||||||||||||
Sbjct  1540044  GCTACCCGTCGCTGCCTTGGTAGGCCATTACCCCACCAACAAGCTGATAGGCCGCGAGAC  1540103

Query  601      GATCCCTGACCGCATAAAGCTTTCCAACCCAACCCATGCAGGCCAGGCTCATATTCGGGA  660
                ||||||||||||||||||||||||||||||||||||||||||||||||||||||||||||
Sbjct  1540104  GATCCCTGACCGCATAAAGCTTTCCAACCCAACCCATGCAGGCCAGGCTCATATTCGGGA  1540163

Query  661      TTACCACCAGTTTCCTGGAGCTATACCGAAGTCAAGGGCACGTTCCTCACGTGTTACTCA  720
                ||||||||||||||||||||||||||||||||||||||||||||||||||||||||||||
Sbjct  1540164  TTACCACCAGTTTCCTGGAGCTATACCGAAGTCAAGGGCACGTTCCTCACGTGTTACTCA  1540223

Query  721      CCCGTTCGCCACTCGTGTACCCCCCGAAAGGGGCCTTACCGTCCGACTT  769
                |||||||||||||||||||||||||||||||||||||||||||||||||
Sbjct  1540224  CCCGTTCGCCACTCGTGTACCCCCCGAAAGGGGCCTTACCGTCCGACTT  1540272
```

Figure S1 The 16S rDNA sequence alignment. Query: *P. freudenreichii 2067,* Subject: *P. freudenreichii CIRM-BIA1*^{T}

Table S1 List of proteins detected by nano-LC-MS/MS from spots with relative abundance fold change ≥1.4 (ANOVA, $p \leq 0.05$) from PPA and WBM media during exponential growth phase. Spots with single identification are in bold. From spots with multiple protein identification those with the highest Mascot identification score are indicated and marked with an asterisk. Proteins identified from the spots with fold change 1.4 are marked gray.

Spot no.	Fold change (PPA/ WBM)	Mw	ID Score (p < 0.05)	No of pept.	Seq. cov. (%)	pI	Locus in reference organism	Name	Annotation	Category
									Amino acid metabolism	
1	1.5	77306	571	6	15%	5.3	PFREUD_22890	NadE	glutamine-dependent NAD(+) synthetase	Metabolism of amino acids and related molecules
3	1.4	65568	592	7	20%	4.86	PFREUD_08920	LeuA1	2-isopropylmalate synthase	Metabolism of amino acids and related molecules
13	1.6	40364	213	2	11%	4.97	PFREUD_18160	SerC	phosphoserine aminotransferase	Metabolism of coenzymes and prosthetic groups
20*	-1.7	36744	387	4	20%	5.16	PFREUD_20100	ASADH	aspartate-semialdehyde dehydrogenase	Metabolism of amino acids and related molecules
									Carbohydrate metabolism	
									Inositol metabolism	
12	2.1	39475	304	8	24%	4.77	PFREUD_19880	Ino1	inositol-1-phosphate synthase	Specific carbohydrate metabolic pathway
21*	-1.7	38657	323	6	19%	4.84	PFREUD_19010	IolE	2-keto-myo-inositol dehydratase	Specific carbohydrate metabolic pathway
									Pyruvate metabolism	
4	2.3	63781	147	4	12%	5.71	PFREUD_16710	D-LDH	d-lactate dehydrogenase	Membrane bioenergetics
7*	1.6	54126	1831	36	78%	5.17	PFREUD_15390	Pyk1	pyruvate kinase 1	Main glycolytic pathways
									Wood Werkman -TCA	
1*	1.5	80242	1411	28	53%	5.29	PFREUD_07650	MutB	Methylmalonyl-CoA mutase large subunit	Metabolism of carbohydrates and related molecules
21	-1.7	35059	141	2	7%	4.95	PFREUD_06860	MDH	malate dehydrogenase	Metabolism of carbohydrates and related molecules
7	1.6	59283	343	4	14%	5.34	PFREUD_18870	PycB	methylmalonyl-CoA carboxyltransferase 5S subunit monomer	Metabolism of carbohydrates and related molecules
									Lactose metabolism	
22	-2.8	35214	180	2	11%	5.37	PFREUD_02620	GalE	UDP-glucose 4-epimerase	Specific carbohydrate metabolic pathway
23*	-1.5	37022	584	8	32%	5.21	PFREUD_19150	Fba1	Fructose-bisphosphate aldolase II	Main glycolytic pathways
									Lipid metabolism	
4*	2.3	59534	1731	41	73%	5.58	PFREUD_12990	GlpA	anaerobic glycerol-3-phosphate dehydrogenase subunit A	Specific carbohydrate metabolic pathway
9*	1.5	45884	1629	29	66%	5.27	PFREUD_12980	GlpB	anaerobic glycerol-3-phosphate dehydrogenase subunit B	Specific carbohydrate metabolic pathway

10	1.5	45884	999	11	40%	5.27	PFREUD_12980	GlpB	anaerobic glycerol-3-phosphate dehydrogenase subunit B	Specific carbohydrate metabolic pathway
10	1.5	43861	989	15	73%	5.07	PFREUD_09410	FabF	3-oxoacyl-ACP synthase	2.4 Metabolism of lipids
11*	1.5	45661	1249	16	46%	4.79	PFREUD_02430	CaiB	crotonobetainyl-CoA:carnitine CoA-transferase	2.4 Metabolism of lipid
13*	1.6	43073	1150	22	58%	4.89	PFREUD_02420	CaiA	putative acyl-CoA dehydrogenase	2.4 Metabolism of lipids
**14**	**1.5**	**31894**	**487**	**9**	**15%**	**4.8**	**PFREUD_09430**	**FabH**	**3-oxoacyl-ACP synthase**	**2.4 Metabolism of lipids**
**15**	**1.4**	**32914**	**1038**	**27**	**65%**	**5.4**	**PFREUD_02450**	**MaoC**	**MaoC acyl dehydratase**	**2.4 Metabolism of lipids**
17*	2.2	28116	396	4	34%	4.59	PFREUD_19890	MmsB	beta-hydroxyacid dehydrogenase	2.4 Metabolism of lipids
17	2.2	28117	326	8	18%	4.6	PFREUD_23160	UgpQ2	glycerophosphoryl diester phosphodiesterase	2.4 Metabolism of lipids
									Membrane bioenergetics	
**5**	**1.4**	**60757**	**1691**	**33**	**65%**	**5.45**	**PFREUD_01830**	**GLS**	**Putative glutamate synthase**	**1.4 membrane bioenergetics (electron transport subunit and ATP synthase)**
**8**	**2.2**	**52579**	**1706**	**30**	**69%**	**4.67**	**PFREUD_10490**	**AtpD**	**ATP synthase subunit beta**	**1.4 Membrane bioenergetics**
**16**	**2.3**	**29633**	**676**	**12**	**51%**	**4.54**	**PFREUD_02490**	**FixB**	**electron transfer flavoprotein FixB**	**1.4 Membrane bioenergetics**
									Transport proteins	
3	1.4	62791	157	2	6%	4.9	PFREUD_07970	ChvD	ABC transporter ATP-binding protein	1.2 Transport/binding proteins and lipoproteins
22*	-2.8	30956	577	17	43%	5.88	PFREUD_01620	SalX	ABC transporter ATP-binding protein	1.2 Transport/binding proteins and lipoproteins
									Protein synthesis	
**2**	**1.5**	**64546**	**709**	**9**	**22%**	**5.16**	**PFREUD_11940**	**GlnRS**	**glutaminyl-tRNA synthetase**	**Aminoacyl-tRNA synthetases**
3*	1.4	64150	1792	24	47%	4.83	PFREUD_14690	ProRS	prolyl-tRNA synthetase	Aminoacyl-tRNA synthetases
3	1.4	76982	445	6	14%	4.83	PFREUD_05640	EF-G	elongation factor G	Translation elongation
**6**	**1.5**	**56027**	**274**	**2**	**20%**	**5.79**	**PFREUD_13090**	**MiaB1**	**2-methylthioadenine synthetase**	**Translation elongation**
10*	1.5	35694	1472	18	71%	5.12	PFREUD_14780	RpsB	30S ribosomal protein S2	Ribosomal proteins
11	1.5	46219	819	7	38%	4.82	PFREUD_14130	CysRS1	cysteinyl-tRNA synthetase	Aminoacyl-tRNA synthetases
11	1.5	39306	233	2	14%	4.78	PFREUD_10360	PrfA	peptide chain release factor 1	Nonribosomal protein synthesis
17	2.2	20647	318	4	20%	4.66	PFREUD_11510	EF-P	elongation factor P	Translation elongation
									Peptidases	
10	1.5	44572	590	6	22%	5.23	PFREUD_18560	AbgB	peptidase, M20/M25/M40 family	Metabolism of amino acids and related molecules
10	1.5	45372	112	2	5%	5.14	PFREUD_16940	ApeB	aminopeptidase	Protein degradation
									Stress proteins	
1	1.5	94317	1003	15	16%	5.33	PFREUD_17920	ClpB2	ATP-dependent Clp protease B2	Protein folding
4	2.3	56504	203	5	13%	4.72	PFREUD_18470	GroL2	molecular chaperone GroEL	Protein folding
19	-1.5	93458	3811	61	71%	5.07	PFREUD_19250	ClpB1	ATP-dependent Clp protease B1	Protein folding
23	-1.5	33620	362	4	22%	5.14	PFREUD_16420	Cys2	cysteine synthase	Metabolism of amino acids and related molecules
**24**	**-1.5**	**33620**	**903**	**16**	**58%**	**5.14**	**PFREUD_16420**	**Cys2**	**cysteine synthase**	**Metabolism of amino acids and related molecules**
**25**	**-1.9**	**33620**	**1557**	**34**	**82%**	**5.14**	**PFREUD_16420**	**Cys2**	**cysteine synthase**	**Metabolism of amino acids and related molecules**

**26**	**-1.5**	**22619**	**392**	**4**	**26%**	**5.28**	**PFREUD_06110**	**SodA**	**Superoxide dismutase**	**Miscellaneous**
**27**	**-1.5**	**22619**	**611**	**16**	**47%**	**5.28**	**PFREUD_06110**	**SodA**	**Superoxide dismutase**	**Miscellaneous**
28*	-2.4	22828	719	13	68%	5.28	PFREUD_06110	SodA	superoxide dismutase	Miscellaneous
									B12 biosynthesis	
13	1.6	35968	740	12	49%	4.89	PFREUD_18910	HemB	delta-aminolevulinic acid dehydratase	Metabolism of coenzymes and prosthetic groups
									Miscellaneous	
**18**	**1.6**	**23335**	**530**	**7**	**44%**	**5.02**	**PFREUD_23970**	**SDR**	**Oxidoreductase**	**Miscellaneous**
20	-1.7	40589	147	2	8%	5.34	PFREUD_19130	AdhC	zinc-containing alcohol dehydrogenase	Miscellaneous
									Other	
9	1.5	46935	174	2	8%	5.3	PFREUD_19280	PurA	adenylosuccinate synthetase	Metabolism of nucleotides and nucleic acids
17	2.2	29040	179	2	15%	4.81	PFREUD_03380	ThiM	Hydroxyethylthiazole kinase	Metabolism of coenzymes and prosthetic groups
20	-1.7	39350	256	3	22%	5.19	PFREUD_21640	CysD	sulfate adenylyltransferase	Metabolism of sulfur
28	-2.4	19697	174	2	15%	5.24	PFREUD_23460	SSB	ssDNA-binding protein	DNA replication
12*	2.1	40517	1261	30	80%	4.75	PFREUD_16140	PF47	protein mrp (ATP-binding protein involved in chromosome partitioning)	Adaptation to atypical conditions

Table S2 List of proteins detected by nano-LC-MS/MS from spots with relative abundance fold-change ≥1.5 (ANOVA, $p \leq 0.05$) from PPA and SWP media during stationary growth phase. Spots with single identification are in bold. From spots with multiple protein identification those with the highest Mascot identification score are indicated and marked with an asterisk

Spot no.	Fold-change (PPA/ SWP)	Mw (Da)	pI	ID score	No. match. pept.	Seq. cov. (%)	Locus in reference organism	Name	Annotation	Category
									Amino acid metabolism	
44*	2.3	52727	5.17	620	7	20	PFREUD_08280	ThrC	threonine synthase	Metabolism of amino acids and related molecules
44	2.3	52087	5.28	274	3	9	PFREUD_18740	GlyA	glycine hydroxymethyltransferase	Metabolism of amino acids and related molecules
48	1.5	48354	5.28	147	2	5	PFREUD_20440	MAO	flavin-containing amine oxidase	Metabolism of amino acids and related molecules
50*	3	39604	4.81	829	34	26	PFREUD_00370	AlaDH	alanine dehydrogenase	Cell wall
51*	2	39604	4.81	804	20	26	PFREUD_00370	AlaDH	alanine dehydrogenase	Cell wall
64*	-1.6	55513	4.95	848	11	27	PFREUD_11910	TrpE	anthranilate synthase component I	Metabolism of amino acids and related molecules
65	-1.5	52727	5.17	411	6	12	PFREUD_08280	ThrC	threonine synthase	Metabolism of amino acids and related molecules
65	-1.5	48354	5.28	201	2	5	PFREUD_20440	MAO	flavin-containing amine oxidase	Metabolism of amino acids and related molecules
71	-	43566	5.53	193	2	7	PFREUD_13960	ArgD	acetylornithine aminotransferase	Metabolism of amino acids and related molecules
73	-2.1	44997	5.13	168	3	7	PFREUD_14000	AlaT	aspartate aminotransferase	Metabolism of amino acids and related molecules
77	-1.8	36744	5.16	196	2	11	PFREUD_20100	ASADH	aspartate-semialdehyde dehydrogenase	Metabolism of amino acids and related molecules
88	-1.6	30334	5.02	659	11	38	PFREUD_11900	TrpC	indole-3-glycerol phosphate synthase	Metabolism of amino acids and related molecules
88	-1.6	30601	4.85	128	2	4	PFREUD_16230	LDC	lysine decarboxylase	Metabolism of amino acids and related molecules
									Aspartate metabolism	
43*	3.6	53385	5.14	825	62	25	PFREUD_16320	AspA1	aspartate ammonia-lyase	Metabolism of amino acids and related molecules
43	3.6	53526	5.12	748	48	26	PFREUD_16330	AspA2	aspartate ammonia-lyase	Metabolism of amino acids and related molecules
45*	2.4	53526	5.12	805	27	28	PFREUD_16330	AspA2	aspartate ammonia-lyase	Metabolism of amino acids and related molecules

45	2.4	53385	5.14	776	28	25	PFREUD_16320	AspA1	aspartate ammonia-lyase	Metabolism of amino acids and related molecules
45	2.4	52812	4.99	145	2	6	PFREUD_01460	ArgG	argininosuccinate synthase	Metabolism of amino acids and related molecules
65	-1.5	48951	5.17	777	9	23	PFREUD_13940	ArgH	argininosuccinate lyase	Metabolism of amino acids and related molecules
									Valine leucine and isoleucine biosynthesis	
76*	-2.1	37335	5.01	819	15	46	PFREUD_13390	IlvC	ketol-acid reductoisomerase	Metabolism of amino acids and related molecules
76	-2.1	40036	5.02	259	3	15	PFREUD_13390	IlvE	branched-subunit amino acid aminotransferase	Metabolism of amino acids and related molecules
									Carbohydrate metabolism	
									Pentose phosphate	
32	2	97159	5.61	239	2	8	PFREUD_12930	Phk	phosphoketolase pyrophosphate	Main glycolytic pathways
33	3	97159	5.61	430	7	10	PFREUD_12930	Phk	phosphoketolase pyrophosphate	Main glycolytic pathways
34	4.2	97159	5.61	650	7	14	PFREUD_12930	Phk	phosphoketolase pyrophosphate	Main glycolytic pathways
44	2.3	55556	5.21	538	9	20	PFREUD_00760	XylB	xylulokinase	Metabolism of carbohydrates and related molecules
65	-1.5	52863	5.25	915	14	20	PFREUD_08090	Gnd2	6-phosphogluconate dehydrogenase	Main glycolytic pathways
66*	-1.5	52863	5.25	1117	14	25	PFREUD_08090	Gnd2	6-phosphogluconate dehydrogenase	Main glycolytic pathways
74*	-1.8	38056	4.84	651	10	36	PFREUD_04970	Tal1	transaldolase	Main glycolytic pathways
87	-3.6	32249	5.4	477	4	31	PFREUD_15070	OpcA	glucose 6-phosphate dehydrogenase effector OpcA	Main glycolytic pathways
69	-1.7	42603	5.44	200	2	10	PFREUD_00690	AckA	acetate kinase	Metabolism of carbohydrates and related molecules
71	-	42603	5.44	196	3	10	PFREUD_00690	AckA	acetate kinase	Metabolism of carbohydrates and related molecules
									Glycolysis	
35	1.9	96275	4.81	1272	12	28	PFREUD_03230	PPDK	pyruvate phosphate dikinase	Metabolism of carbohydrates and related molecules
50	3	43333	4.94	798	11	35	PFREUD_12040	PFP	pyrophosphate phosphofructokinase	Carbohydrate metabolism
51	2	43333	4.94	531	8	27	PFREUD_12040	PFP	pyrophosphate phosphofructokinase	Carbohydrate metabolism
62	-1.9	63416	4.88	417	6	16	PFREUD_23740	AglA	alpha-1,4-glucosidase	Specific carbohydrate metabolic pathway (galactose)
63	-	58031	5.05	265	2	9	PFREUD_19470	PtsA	PTS enzyme I	Protein of unknown function, without similarity to other proteins (carbohydrate transport and metabolism)
67*	-1.8	42524	5.21	1217	23	48	PFREUD_15120	PGK	phosphoglycerate kinase	Main glycolytic pathways

68	-1.5	42524	5.21	251	3	14	PFREUD_15120	PGK	phosphoglycerate kinase	Main glycolytic pathways
74	-1.8	34225	4.91	413	5	19	PFREUD_12840	L-LDH	L-lactate dehydrogenase	Main glycolytic pathways
**75**	**-3**	**34225**	**4.91**	**661**	**15**	**31**	**PFREUD_12840**	**L-LDH**	**L-lactate dehydrogenase**	**Main glycolytic pathways**
77*	-1.8	36261	5.26	1401	48	65	PFREUD_15130	GAPDH	glyceraldehyde-3-phosphate dehydrogenase	Metabolism of coenzymes and prosthetic groups
**78**	**-1.6**	**35532**	**5.17**	**609**	**8**	**27**	**PFREUD_15130**	**GAPDH**	**glyceraldehyde-3-phosphate dehydrogenase**	**Metabolism of coenzymes and prosthetic groups**
79*	-1.8	35532	5.17	667	7	34	PFREUD_15130	GAPDH	glyceraldehyde-3-phosphate dehydrogenase	Metabolism of coenzymes and prosthetic groups
79	-1.8	35795	XX	XX	11	33	PFREUD_02200	BkdA2	2-oxoisovalerate dehydrogenase subunit beta	Metabolism of amino acids and related
80	-2.1	32398	5.51	415	5	25	PFREUD_22330	YeaD	aldose 1-epimerase	Metabolism of carbohydrates and related molecules
**81**	**-2**	**32368**	**5.02**	**582**	**8**	**34**	**PFREUD_23890**	**FBA2**	**Fructose-bisphosphate aldolase class I**	**Main glycolytic pathways**
82*	-2.4	37022	5.21	525	10	27	PFREUD_19150	FBA1	Fructose-bisphosphate aldolase class II	Main glycolytic pathways
83	-3.1	37022	5.21	180	2	8	PFREUD_19150	FBA1	Fructose-bisphosphate aldolase class II	Main glycolytic pathways
84*	-1.6	37022	5.14	861	24	16	PFREUD_19150	FBA1	Fructose-bisphosphate aldolase class II	Main glycolytic pathways
84	-1.6	35795	5.21	174	2	38	PFREUD_02200	BkdA2	2-oxoisovalerate dehydrogenase subunit beta	Metabolism of amino acids and related
87*	-3.6	35532	5.17	1032	10	54	PFREUD_15130	GAPDH	glyceraldehyde-3-phosphate dehydrogenase	Metabolism of coenzymes and prosthetic groups
88	-1.6	32368	5.02	521	6	29	PFREUD_23890	FBA2	Fructose-bisphosphate aldolase class I	Main glycolytic pathways
89*	-1.5	27830	5.05	424	5	26	PFREUD_15110	TIM1	triosephosphate isomerase 1	Main glycolytic pathways
									Wood Werkman-TCA	
**29**	**1.6**	**137778**	**5.22**	**2689**	**40**	**33**	**PFREUD_01840**	**NifJ1**	**pyruvate synthase/pyruvate-flavodoxin oxidoreductase**	**Membrane bioenergetics**
**30**	**1.9**	**137778**	**5.22**	**2525**	**59**	**34**	**PFREUD_01840**	**NifJ1**	**pyruvate synthase/pyruvate-flavodoxin oxidoreductase**	**Membrane bioenergetics**
**31**	**1.7**	**137778**	**5.22**	**3124**	**71**	**42**	**PFREUD_01840**	**NifJ1**	**pyruvate synthase/pyruvate-flavodoxin oxidoreductase**	**Membrane bioenergetics**
**36**	**1.7**	**96275**	**4.81**	**2351**	**50**	**39**	**PFREUD_03230**	**PPDK**	**pyruvate phosphate dikinase**	**Metabolism of carbohydrates and related molecules**
**39**	**2.4**	**75276**	**6**	**1488**	**36**	**36**	**PFREUD_14311**	**SdhA3**	**succinate dehydrogenase flavoprotein subunit**	**Membrane bioenergetics**
40*	1.9	75276	6	1227	21	31	PFREUD_14310	SdhA3	succinate dehydrogenase flavoprotein subunit	Membrane bioenergetics
41	1.7	66057	5.22	373	4	10	PFREUD_18860	MmdA	methylmalonyl-CoA carboxyltransferase subunit 12S monomer	Specific carbohydrate metabolic pathway

45	2.4	53022	5	200	1	6	PFREUD_19100	IolA	myo-inositol catabolism protein IolA	Specific carbohydrate metabolic pathway
45	2.4	57987	5.97	184	2	6	PFREUD_18870	PycB	methylmalonyl-CoA carboxyltransferase 5S subunit monomer	Metabolism of carbohydrates and related
46*	2	49814	5.08	483	5	22	PFREUD_10890	LPD	dihydrolipoamide dehydrogenase	Metabolism of carbohydrates and related molecules
48*	1.5	51435	5.34	897	56	39	PFREUD_16300	FumC	fumarate hydratase class II	TCA cycle
49	1.6	48580	5.67	276	3	14	PFREUD_01090	GltA2	citrate synthase	TCA cycle
53	1.9	27939	5.42	117	0	9	PFREUD_09260	SdhB	succinate dehydrogenase subunit B	Membrane bioenergetics
55*	2.8	27939	5.42	269	3	18	PFREUD_09260	SdhB	succinate dehydrogenase subunit B	Membrane bioenergetics
64	-1.6	49814	5.08	466	7	19	PFREUD_10890	LPD	dihydrolipoamide dehydrogenase	Metabolism of carbohydrates and related molecules
65	-1.5	51435	5.34	310	2	17	PFREUD_16300	FumC	fumarate hydratase class II	TCA cycle
66	-1.5	51435	5.34	420	5	18	PFREUD_16300	FumC	fumarate hydratase class II	TCA cycle
67	-1.8	47181	5.29	628	6	24	PFREUD_09540	GabT	**4-aminobutyrate aminotransferase**	Metabolism of amino acids and related molecules
68	-1.5	47181	5.29	719	9	29	PFREUD_09540	GabT	**4-aminobutyrate aminotransferase**	Metabolism of amino acids and related molecules
80	-2.1	31447	5.22	504	5	28	PFREUD_16110	CitE	citrate lyase subunit beta	TCA cycle
87	-3.6	31447	5.22	220	2	9	PFREUD_16110	CitE	citrate lyase subunit beta	TCA cycle
									Lipid metabolism	
51	2	45661	4.79	123	2	4	PFREUD_02430	CaiB	crotonobetainyl-CoA:carnitine CoA-transferase	Metabolism of lipids
56	2	25770	4.72	132	2	8	PFREUD_02480	FixA	electron transfer flavoprotein FixA	Membrane bioenergetics (electron transport subunit and ATP synthase
61*	-2.3	61241	5.08	806	9	24	PFREUD_23780	CaiC	Fatty-acyl-CoA synthase	Metabolism of lipids
									Metabolism of coenzymes and prosthetic groups	
52	1.5	39443	5.22	176	2	8	PFREUD_06270	PanK	pantothenate kinase	Metabolism of coenzymes and prosthetic groups
52	1.5	41862	5.19	175	2	7	PFREUD_10010	BioB	biotin synthase	Metabolism of coenzymes and prosthetic groups
73	-2.1	41862	5.19	161	2	7	PFREUD_10010	BioB	biotin synthase	Metabolism of coenzymes and prosthetic groups
77	-1.8	36833	5.26	473	7	25	PFREUD_07540	MenB	naphthoate synthase	Metabolism of coenzymes and prosthetic groups
82	-2.4	31387	5.14	181	2	11	PFREUD_15040	PdxS	pyridoxal biosynthesis lyase PdxS	Metabolism of coenzymes and prosthetic groups
88*	-1.6	31387	5.14	696	12	34	PFREUD_15040	PdxS	pyridoxal biosynthesis lyase PdxS	Metabolism of coenzymes and prosthetic groups

88	-1.6	32235	5.05	291	3	19	PFREUD_12810	KPR	2-dehydropantoate 2-reductase	Metabolism of coenzymes and prosthetic groups
83	-3.1	30812	5.23	184	2	11	PFREUD_06840	FolD	methylenetetrahydrofolate dehydrogenase (bifunctional protein FolD)	Metabolism of coenzymes and prosthetic groups
84	-1.6	30812	5.29	177	2	14	PFREUD_06840	FolD	methylenetetrahydrofolate dehydrogenase (bifunctional protein FolD)	Metabolism of coenzymes and prosthetic groups
									Membrane bioenergetics	
32*	2	10170 7	5.68	1185	22	22	PFREUD_04320	GlcD/ GlpC	FAD-linked oxidase multidomain	Membrane bioenergetics (electron transport subunit and ATP synthase)
33*	3	10170 7	5.68	1780	36	28	PFREUD_04320	GlcD/ GlpC	FAD-linked oxidase multidomain	Membrane bioenergetics (electron transport subunit and ATP synthase)
34*	4.2	10170 7	5.68	1727	38	29	PFREUD_04320	GlcD/ GlpC	FAD-linked oxidase multidomain	Membrane bioenergetics (electron transport subunit and ATP synthase)
41*	1.7	60757	5.45	1613	41	47	PFREUD_01830	GLS	Putative glutamate synthase	Membrane bioenergetics
**42**	**1.9**	**60757**	**5.45**	**1966**	**67**	**62**	**PFREUD_01830**	**GLS**	**Putative glutamate synthase**	**Membrane bioenergetics**
**47**	**1.5**	**47953**	**5.7**	**678**	**11**	**27**	**PFREUD_05140**	**GGR**	**Electron transfer oxidoreductase (Geranylgeranyl reductase superfamily)**	**Membrane bioenergetics**
49*	1.6	49297	5.72	1432	36	48	PFREUD_05210	NuoF	NADH dehydrogenase I subunit F	Membrane bioenergetics (electron transport subunit and ATP synthase
58	-1.8	85720	5.1	185	1	5	PFREUD_05220	NuoG	NADH dehydrogenase I subunit G	Membrane bioenergetics (electron transport subunit and ATP synthase)
63*	-	61453	5.06	1182	20	36	PFREUD_10470	AtpA	ATP synthase subunit alpha	Membrane bioenergetics (electron transport subunit and ATP synthase)
									Transport proteins	
53	1.9	28958	5.4	281	6	18	PFREUD_14840	FepC	ABC transporter ATP-binding protein (ABC-type cobalamin/Fe3+-siderophores transport systems)	Transport/binding proteins and lipoproteins
62	-1.9	61661	5.06	932	13	27	PFREUD_05930	DdpA	ABC transporter substrate-binding protein	Transport/binding of proteins/peptides
87	-3.6	29828	5.26	513	5	35	PFREUD_19980	PstB	phosphate ABC transporter substrate-binding protein	Transport/binding of inorganic ions
87	-3.6	31039	5.45	291	3	19	PFREUD_10060	DppF	ABC transporter ATP-binding protein (ABC-type dipeptide/oligopeptide/nickel transport system)	Transport/binding of inorganic ions
80	-2.1	36077	5.35	172	3	8	PFREUD_07640	ArgK	kinase	Transport/binding of amino-acids

									Cell wall biogenesis	
38	2.6	78318	4.94	180	2	5	PFREUD_19400	PBP-1A	penicillin-binding protein	Cell wall
68*	-1.5	45318	5.29	886	14	41	PFREUD_00570	UGM	UDP-galactopyranose mutase	Cell wall
74	-1.8	40727	4.85	472	5	21	PFREUD_13250	DdlA	D-alanine--D-alanine ligase	Cell wall
									DNA recombination and repair	
52	1.5	39046	5.11	365	5	18	PFREUD_20160	DisA	DNA integrity scanning protein DisA	DNA recombination, and repair
73	-2.1	39046	5.11	398	6	18	PFREUD_20160	DisA	DNA integrity scanning protein DisA	DNA recombination, and repair
76	-2.1	36678	4.96	183	2	12	PFREUD_11700	RuvB	Holliday junction ATP-dependent DNA helicase	DNA recombination, and repair
									Transcription regulation	
53	1.9	26294	5.33	103	1	9	PFREUD_01400	OmpR	transcriptional regulator	Transcription regulation
79	-1.8	28725	5.26	309	4	18	PFREUD_15890	PspA	phage shock protein A	Transcription regulation
88	-1.6	29867	5	570	7	37	PFREUD_23070	RpiR	RpiR family transcriptional regulator	Transcription regulation
									Protein synthesis	
35*	1.9	90689	4.74	1610	20	33	PFREUD_14010	PheRS	phenylalanyl-tRNA synthetase subunit beta	Aminoacyl-tRNA synthetases
38*	2.6	75867	4.88	1555	35	33	PFREUD_06230	EF-G	GTP phosphohydrolase, elongation factor G	Translation elongation
**46**	**2**	**47518**	**4.99**	**270**	**4**	**11**	**PFREUD_01350**	**SerRS1**	**seryl-tRNA synthetase**	**Aminoacyl-RNA synthetases**
									Peptidases	
44	2.3	53283	5.2	363	3	14	PFREUD_16090	PepP	Xaa-Pro aminopeptidase	Protein degradation
65*	-1.5	53283	5.2	1080	15	34	PFREUD_16090	PepP	Xaa-Pro aminopeptidase	Protein degradation
66	-1.5	53283	5.2	975	13	33	PFREUD_16090	PepP	Xaa-Pro aminopeptidase	Protein degradation
72*	-2.4	42925	5	946	22	33	PFREUD_00340	AbgB	amidohydrolase	Protein degradation
73	-2.1	39184	5.31	327	3	19	PFREUD_08810	PIP2	proline iminopeptidase	Protein degradation
74	-1.8	33462	4.8	132	1	5	PFREUD_16060	MAP	methionine aminopeptidase	Protein degradation
									Selenocompound metabolism	
51	2	45129	4.93	593	7	20	PFREUD_22880	CBL	cystathionine beta-lyase	Metabolism of amino acids and related molecules
52	1.5	41688	5.17	283	3	13	PFREUD_18590	CGS	cystathionine gamma-synthase	Metabolism of amino acids and related molecules
62*	-1.9	62897	4.68	1023	11	33	PFREUD_06960	MetRS	methionyl-tRNA synthetase	Aminoacyl-tRNA synthetases

69*	-1.7	45374	5.44	884	11	36	PFREUD_14940	SufS	cysteine desulfurase	Transport/binding proteins and lipoproteins
71*	-	45374	5.44	946	9	36	PFREUD_14940	SufS	cysteine desulfurase	Transport/binding proteins and lipoproteins
73*	-2.1	41688	5.17	411	8	18	PFREUD_18590	CGS	cystathionine gamma-synthase	Metabolism of amino acids and related molecules
73	-2.1	39350	5.19	255	2	17	PFREUD_21640	CysD	sulfate adenylyltransferase	Metabolism of sulfur
									Pirimidine metabolism	
72	-2.4	42817	5.08	396	4	14	PFREUD_11480	CarA	carbamoyl-phosphate synthase small subunit	Transport/binding of nucleosides, nucleotides, purines and pyrimidines
77	-1.8	37599	5.18	316	4	12	PFREUD_17440	PyrB	aspartate carbamoyltransferase	Metabolism of nucleotides and nucleic acids
									Stress proteins	
**37**	**2.3**	**94317**	**5.33**	**2756**	**48**	**44**	**PFREUD_17920**	**ClpB2**	**ATP-dependent Clp protease B2**	**Protein folding**
58*	-1.8	93458	5.07	2390	38	42	PFREUD_19250	ClpB1	ATP-dependent Clp protease B1	Protein folding
**59**	**-2**	**93458**	**5.07**	**2766**	**46**	**50**	**PFREUD_19250**	**ClpB1**	**ATP-dependent Clp protease B1**	**Protein folding**
**60**	**-1.5**	**93458**	**5.07**	**2527**	**49**	**46**	**PFREUD_19250**	**ClpB1**	**ATP-dependent Clp protease B1**	**Protein folding**
68	-1.5	43958	5.3	480	7	23	PFREUD_22290	MshA	group 1 glycosyl transferase (D-inositol-3-phosphate glycosyltransferase)	Cell wall (mycothiol biosynthesis)
**70**	**-2**	**49226**	**5.5**	**1290**	**20**	**51**	**PFREUD_17610**	**GDH**	**glutamate dehydrogenase**	**Metabolism of amino acids and related molecules**
71	-	49226	5.5	120	2	4	PFREUD_17610	GDH	glutamate dehydrogenase	Metabolism of amino acids and related molecules
79	-1.8	33620	5.14	231	2	16	PFREUD_16420	Cys2	cysteine synthase A	Metabolism of amino acids and related molecules
80*	-2.1	33620	5.14	650	17	33	PFREUD_16420	Cys2	cysteine synthase A	Metabolism of amino acids and related molecules
82	-2.4	33620	5.14	418	5	21	PFREUD_16420	Cys2	cysteine synthase A	Metabolism of amino acids and related molecules
83*	-3.1	33620	5.14	522	5	35	PFREUD_16420	Cys2	cysteine synthase A	Metabolism of amino acids and related molecules
84	-1.6	33620	5.14	299	3	13	PFREUD_16420	Cys2	cysteine synthase A	Metabolism of amino acids and related molecules
**85**	**-3.7**	**33620**	**5.14**	**431**	**6**	**28**	**PFREUD_16420**	**Cys2**	**cysteine synthase A**	**Metabolism of amino acids and related molecules**
**86**	**-**	**33620**	**5.14**	**826**	**47**	**54**	**PFREUD_16420**	**Cys2**	**cysteine synthase A**	**Metabolism of amino acids and related molecules**
87	-3.6	33620	5.14	842	11	35	PFREUD_16420	Cys2	cysteine synthase A	Metabolism of amino acids and related molecules
90*	-1.7	26907	5.18	849	17	58	PFREUD_14950	SufC	ABC transporter ATPase	Transport/binding proteins and lipoproteins

**92**	**-1.5**	**22619**	**5.28**	**314**	**6**	**18**	**PFREUD_06110**	**SodA**	**Superoxide dismutase**	**Miscellaneous**
**93**	**-5.2**	**16830**	**4.93**	**575**	**8**	**58**	**PFREUD_09500**	**IbpA**	**Molecular chaperone (small heat shock protein)**	**Protein folding**
									B_{12} biosynthesis	
65	-1.5	49597	5.19	764	11	31	PFREUD_19260	HemL2	glutamate-1-semialdehyde 2,1-aminomutase	**Metabolism of coenzymes and prosthetic groups**
66	-1.5	49597	5.19	949	15	37	PFREUD_19260	HemL2	glutamate-1-semialdehyde 2,1-aminomutase	**Metabolism of coenzymes and prosthetic groups**
72	-2.4	46227	5.1	216	2	11	PFREUD_18880	HemL1	glutamate-1-semialdehyde 2,1-aminomutase	**Metabolism of coenzymes and prosthetic groups**
89	-1.5	26867	4.91	404	7	34	PFREUD_07680	CbiL	CbiL Precorrin-2 C20-methyltransferase	**Metabolism of coenzymes and prosthetic groups**
90	-1.7	25015	5.22	260	3	25	PFREUD_05110	CbiO	ABC transporter ATP-binding protein	Transport/binding proteins and lipoproteins
									Miscellaneous	
53*	1.9	29476	5.56	311	8	17	PFREUD_18650	GlpC	Oxidoreductase	Miscellaneous
54*	2	29476	5.56	507	14	34	PFREUD_18650	GlpC	Oxidoreductase	Miscellaneous
55	2.8	29476	5.56	156	3	9	PFREUD_18650	GlpC	Oxidoreductase	Miscellaneous
**57**	**1.6**	**24018**	**5.18**	**352**	**21**	**33**	**PFREUD_22440**	**SDR_c**	**Short subunit dehydrogenase (classical)**	**Miscellaneous**
78*	-1.6	37025	5.36	755	12	37	PFREUD_13440	Kv	aldo/keto reductase (voltage-dependent potassium channel beta subunit)	Miscellaneous
87	-3.6	31191	5.49	685	9	39	PFREUD_03160	DkgA	aldo/keto reductase (2,5-diketo-D-gluconate reductase A)	Miscellaneous
**91**	**-1.8**	**23335**	**5.02**	**476**	**6**	**46**	**PFREUD_23970**	**SDR**	**Oxidoreductase**	**Miscellaneous**
									Other	
40	1.9	68281	5.95	404	6	15	PFREUD_21620	CysI	Sulfite reductase [ferredoxin]	2.7 Metabolism of sulfur
50	3	42499	5.17	212	2	10	PFREUD_03730	Csb1-like	CRISPR-associated protein with similarity to Csb1	6. Protein of unknown function, without similarity to other proteins
51	2	43375	4.87	306	3	18	PFREUD_03120	DUF1015	Protein of unknown function (DUF1015)	Protein of unknown function similar to proteins from other organisms
53	1.9	27851	5.4	190	2	12	PFREUD_16080	COG1579	Zn-ribbon protein, possibly nucleic acid-binding [General function prediction only	Protein of unknown function similar to proteins from other organisms
54	2	27851	5.4	439	6	36	PFREUD_16080	COG1579	Zn-ribbon protein, possibly nucleic acid-binding [General function prediction only	Protein of unknown function similar to proteins from other organisms
56*	2	23227	4.7	804	34	52	PFREUD_18630	COG1556	Uncharacterized conserved protein [Function unknown]	Protein of unknown function similar to proteins from other organisms
61	-2.3	62746	5.05	575	7	17	PFREUD_20030	ArsA	arsenic ABC transporter ATPase	Detoxification

74	-1.8	36506	4.78	251	2	13	PFREUD_15430	-	Protein of unknown function	Protein of unknown function similar to proteins from other organisms
76	-2.1	39568	5.13	139	2	6	PFREUD_11600	-	Protein of unknown function, highly conserved in Propionibacteria	Protein of unknown function similar to proteins from other organisms
