# Supplementary figures and images for: Food-Like Growth Conditions Support Production of Active Vitamin B12 by Propionibacterium freudenreichii 2067 without DMBI, the Lower Ligand Base, or Cobalt Supplementation
Source: Front Microbiol. 2017 Mar 8;8:368. doi: 10.3389/fmicb.2017.00368 (PMC5340759; doi:10.3389/fmicb.2017.00368)

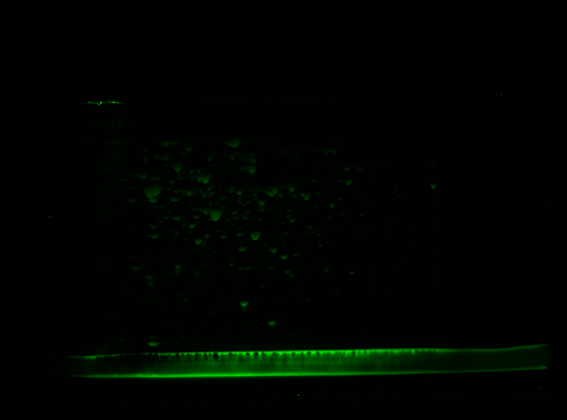

Supplement: Supplementary file 2 [file Image1.TIF]

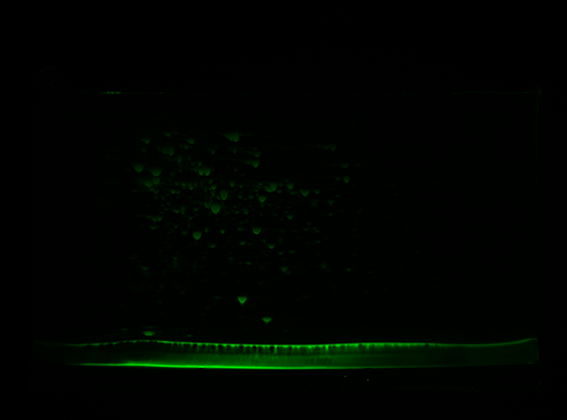

Supplement: Supplementary file 3 [file Image2.TIF]

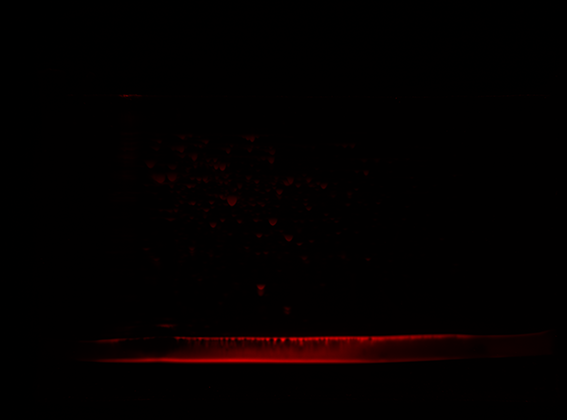

Supplement: Supplementary file 4 [file Image3.TIF]

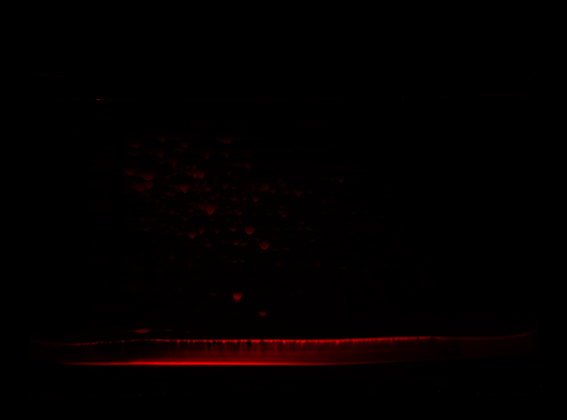

Supplement: Supplementary file 5 [file Image4.TIF]
